# Challenges and Insights in *Aggregatibacter aphrophilus* endocarditis: a review of literature

**DOI:** 10.47487/apcyccv.v4i3.306

**Published:** 2023-09-30

**Authors:** Nathalie Victoria Zacarías Mendoza, Norma Nicole Gamarra Valverde, Víctor Justo Robles Velarde

**Affiliations:** 1 Facultad de Medicina Alberto Hurtado, Universidad Peruana Cayetano Heredia. Lima, Perú. Universidad Peruana Cayetano Heredia Facultad de Medicina Alberto Hurtado Universidad Peruana Cayetano Heredia Lima Peru; 2 Servicio de Cirugía Cardiovascular, Instituto Nacional de Cardiología. Lima, Perú. Servicio de Cirugía Cardiovascular Instituto Nacional de Cardiología Lima Perú

**Keywords:** Bacterial Endocarditis, Gram-negative Bacteria, Aggregatibacter aphrophilus, Endocarditis Bacteriana, Bacterias Gramnegativas, Aggregatibacter aphrophilus

## Abstract

Infective endocarditis is a serious disease associated with high mortality despite recent advances in diagnosis and treatment. *Aggregatibacter aphrophilus* is a fastidious Gram-negative member of the HACEK organisms (*Haemophilus* spp., *Aggregatibacter actinomycetemcomitans*, *Cardiobacterium hominis*, *Eikenella corrodens*, and *Kingella kingae*). *A. aphrophilus* is associated with dental infections but has also been implicated in cases of infective endocarditis. We highlight the importance of a high index of suspicion in symptomatic patients with an initial negative blood culture, particularly in high-risk groups such as patients with congenital valve disease and prosthetic valve. The knowledge of this rare entity may lead to early diagnosis and appropriate management. We review the main characteristics of *Aggregatibacter aphrophilus* endocarditis reported in the medical literature.

## RESUMEN

La endocarditis infecciosa es una enfermedad grave que está asociada con una alta mortalidad a pesar de los avances recientes en el diagnóstico y tratamiento. *Aggregatibacter aphrophilus* es un miembro Gram-negativo de los organismos HACEK (*Haemophilus* spp., *Aggregatibacter actinomycetemcomitans*, *Cardiobacterium hominis*, *Eikenella corrodens* y *Kingella kingae*). *A. aphrophilus* está relacionado con infecciones dentales, pero también ha estado implicado en casos de endocarditis infecciosa. Se destaca la importancia de tener un alto índice de sospecha en pacientes sintomáticos con un cultivo sanguíneo inicial negativo, especialmente en grupos de alto riesgo como pacientes con enfermedad valvular congénita y válvula protésica. El conocimiento de esta entidad poco común puede llevar a un diagnóstico temprano y un manejo adecuado. Revisamos las principales características de la endocarditis por *Aggregatibacter aphrophilus* reportadas en la literatura médica.

## Introduction

*Aggregatibacter aphrophilus* is a member of the HACEK organisms (Haemophilus spp., *Aggregatibacter actinomycetemcomitans*, *Cardiobacterium hominis*, *Eikenella corrodens*, and *Kingella kingae*). *A. aphrophilus* is a fastidious Gram-negative associated with dental infections but has also been implicated in cases of infective endocarditis ^1^.

HACEK endocarditis is a rare disease with an excellent prognosis and simple management if the organism is properly identified. Due to the difficulty of *Aggregatibacter aphrophilus* isolation, this bacterium is rarely seen in blood cultures ^2^. In this paper, we review the main characteristics of *Aggregatibacter aphrophilus* endocarditis reported in the medical literature.

## Literature review

We reviewed PubMed® for cases of *Aggregatibacter aphrophilus* endocarditis. We used the MeSH database to search the terms “infective endocarditis” and “*Aggregatibacter aphrophilus*” in order to increase the sensibility and specificity of the search. The 20 cases with the most significant data are summarized in Table 1. The articles were reviewed to gather information about patient demographics, preexisting heart diseases, and treatment options. In total, 91 studies were identified, of which 20 met the inclusion criteria, describing a total of 20 patients **(**Table 1**)**. The identified studies were performed between 2002 and 2021.

**Table 1:** Main characteristics of patients at hospital admission from the 20 cases of *Aggregatibacter aphrophilus* endocarditis reported in the medical literature. (*Continues on next page*)

Case No. (Reference citation)	Age(y)/sex	Initial clinical presentation	Risk factors	Extra-cardiac Complications	Diagnostic test	Images					Antibiotics and duration	Surgery	Death
						Exams	Images findings	Type of Valve infected	Size of Veg	Veg localization						
1_^(3)^_ 2013	22/M	Nausea, headache and exhaustion	-	-	Blood culture (+)	TTE (-) TEE (+)	Veg on MV	Native	5×4 mm	MV	CRO, 4 weeks.	No	No
2_^(4)^_ 2016	71/M	Fever for 2 weeks.	History of 2 AVR due to IE and a subsequent fail bioprosthetic valve.	No	Blood culture (+)	2D-TEE (-), 2D- TTE (-), 3D-TTE (+)	Veg on prosthetic AV	Biological p.	15 mm	Prosthetic AV	NA, 2 weeks.	AVR	No
3 _^(5)^_ 2021	32/M	Pyrexia, dyspnea and HF	History of AVR due to BAV.	No	16S rRNA gene sequencing (+), Blood culture (-), Valve culture (-)	TEE (+)	Severe AR with ARA complicated by perforation into the RV.	Biological p.	No	-	CRO, NA.	Debridement, AVR, annular reconstruction and graft replacement of the ascending aorta.	No
4 _^(6)^_ 2017	47/M	Intermittent fever, chills, and decreased urine output for 2 weeks. Systolic murmur IV/VI	No	Roth’s spots	16S rRNA polymerase chain reaction (PCR) and sequencing, Blood culture (-)	TEE (+)	Veg on MV	Native	NA	MV	CRO + VAN + CIP + teicoplanin + daptomycin + ertapenem, NA.	MVR and ring annuloplasty	No
5 _^(7)^_ 2021	51/M	General malaise, vomiting, diarrhea, fever, sweats and myalgia for 2 weeks.	No	Embolic stroke and digital infarction.	16S rRNA sequencing, Blood culture (-)	TTE (+)	Moderate AR	Native	No	No	CRO, 6 weeks.	No	No
6 _^(8)^_ 2017	72/F	Persistent high fever and acute renal failure	No	Glomerulonephritis, positive PR3-ANCA, cerebral embolism and hemorrhage	br-PCR and sequencing (+), Blood culture (-)	TTE (+)	Severe MR and veg on MV	Native	1 cm	MV	CRO, 4 weeks	MVR	No
7 _^(9)^_ 2014	62/M	Fever, chills, night sweats, fatigue, and ten-pound weight loss over a four-month period. Systolic murmur and JVD	Dual-chamber pacemaker placement in 1996 for complete heart block with subsequent lead manipulation in 2007	No	Blood culture (+)	TEE (+)	Veg on TV and on the RV pacemaker lead	Native	TV (1.2 cm × 0.7 cm) and pacemaker lead (NA)	Native and right ventricular pacemaker lead.	CRO, 6 weeks.	Device removal and temporary jugular venous pacing wires were placed	No
8 _^(10)^_ 2021	25/M	Fever, myalgia and a non-productive cough.	Congenital heart disease with a true BAV and ascending aortopathy	No	Blood culture (+)	TTE (+)	ARA	No	No	No	CRO + GEN, NA	Debridement and redo Bentall operation with a mechanical AV and replacement of the RV to PA conduit	No
9 _^(11)^_ 2017	65/F	Left hemiparesis, frontal and nasal headaches, rotational vertigo when getting up, afebrile. Grade 2/6 systolic murmur.	15 years: rheumatic fever. 51 years: 2 AVR and MVR	Cerebral embolism	Blood culture (+) 3/6	TTE (-) TEE (+) PET/CT (+)	Veg on MV, Veg on AV	Mechanical p.	6 x 5 mm	Prosthetic MV and Prosthetic AV	CRO, 6 weeks	No	No
10 _^(12)^_ 2022	74/M	Persistent pain in the right shoulder, general weakness, chills, palpitations and lack of appetite. 98.6° F temperature.	No	No	Blood culture (+) 1/6	TEE (+) TTE (+)	Severe MR and veg on MV	Native	NA	MV	CRO, 3 weeks	No	No
11 _^(13)^_ 2013	61/M	Lethargy, night sweats, fever of 100° F, decreased appetite, and erratic low blood glucose without weight loss.	History of AVR due to BAV. Diabetes	Splinter hemorrhage in one finger	Blood culture (+) 7/8	TEE (+) TTE (+)	Veg on MV	Protease valve	No	Anterior mitral leaflet	CRO, 4 weeks + GEN, 2 weeks	No	No
12 ^14^ 2019	53/M	Confusion, fever, night sweats, chills, and an unintentional twenty-pound weight loss over the past two months.	No	No	Blood culture (-)	TEE (+) TTE (+)	Large weakly echogenic MV veg.	Native	1.5 x 1.0 cm	Posterior mitral valve	CRO, 6 weeks	MVR with bioprosthetic valve	No
13 ^(^^15^ 2016	56/M	Headache, acute decrease in psychomotor function, and fever of 40° C.	9 years: closure of ASDs by patent foramen ovale with surgical patch.	Cerebral abscess	Blood culture (-)	TTE (-) TEE (+)	High right left shunt confirming dehiscence of the surgical patch closure of ASDs.	NA	NA	No	CRO, 8 weeks + RF, 8 weeks.	Surgical patch removal	No
14 ^16^ 2018	12/F	Fatigue, weight loss, intermittent fever, chills, cough and night sweats. Pan-systolic II/VI heart murmur.	Perimembranous VSD.	No	Blood culture (+) 2/2	TTE (+)	Large irregularly bordered echogenic mass attached to the right atrial side of TV. MVR.	Native	3 cm x 2.5 cm	TV	MEM + DO + GEN, NA.	-	NA
15 ^(^^17^ 2017	42/M	Fever and jaundice.	17 years: MVR due to rheumatic fever.	Cerebral embolism	Blood culture (+) 1/1, 16S rRNA sequencing (+)	TTE (-) TEE (+)	Thrombus and veg in mechanical MV.	Protease valve	NA	No	CRO, 8 weeks.	The infected mitral valve prosthesis and the left upper pulmonary vein thrombus were removed	No
16 ^18^ 2009	42/F	Fatigue and discomfort on the left side of the chest, vomiting, and increasing lethargy. Reduced level of consciousness and urinary incontinence. Fever of 40.2° C	Nicotine and alcohol abuse	Bilateral infarction of the cerebral arteries	Blood culture (-)	TEE (+)	Mobile veg on the noncoronary cusp of the aortic valve.	Native	NA	No	CRO + VA.	No	No
17 ^(^^19^ 2015	4/F	Signs of heart failure	2 years: Contegra D-valved conduit (CVC) placement - transposition of the great arteries with VSD and PS.	No	Blood culture (-)	TTE (+)	Veg on the pulmonary side of the prosthetic valved conduit	Protease valve	4 × 5 mm	No	CRO	No	No
18 ^20^ 2012	58/M	Left flank pain followed by fever with chills for 2 weeks. Systolic murmur (Grade 3)	Congenital valvular heart disease	Spleen abscess	Blood culture (+) 2/2	TTE (+)	Severe AR and a flail Mv with severe MR.	Native	No	No	CRO, 2 weeks	No	No
19 ^(^^21^ 2002	62/M	Fevers lasting 1 week with rigors and diaphoresis. A grade 3/6 systolic crescendo-decrescendo ejection murmur and a soft 1/4 blowing diastolic murmur	Calcific aortic stenosis due to a congenital BAV. Poor dentition	No	Blood culture (+) 2/3	TTE (+) TEE (+)	BAV with moderate AS with mild AR.	Native	No	No	CRO + GEN, 5 days.	No	No
20 ^22^^)^ 2002	25/M		Aortic valvuloplasty at 8 years for correction of congenital AS. Previous dental work with endocarditis prophylaxis. Pierced tongue (2 months before).	No	Blood culture (+) 2/2	No	No	Native	No	No	No	AVR	No

MV: Mitral valve; AV: Aortic valve; AR: Aortic regurgitation; ARA: Aortic Root Abscess; RV: Right ventricle; ASDs: Atrial septal defects; TTE: transthoracic echocardiogram; TEE: Transesophageal echocardiogram; 2D-TEE: 2D Transesophageal echocardiogram; 2D-TTE: 2D transthoracic echocardiogram; 3D-TTE: 3D transthoracic echocardiogram; PET-CT: Positron emission tomography; TV: Tricuspid valve; MR: Mitral regurgitation; MVR: Mitral valve replacement; AVR: Aortic valve replacement; BAV: Balloon aortic valvuloplasty; VSD: Ventricular septal defect; PS: Pulmonic stenosis; AS: Aortic stenosis; JVD: Jugular venous distension; HF: heart failure; CRO: Ceftriaxone; CIP: Ciprofloxacin; VAN: Vancomycin; GEN: Gentamycin; FOF: Fosfomycin; AMP: ampicillin; MEM: Meropenem; DO: Doxycycline.

### Demographics

We have reviewed 20 cases (15 men (75%) and 5 women (25%)); median age: 46,8 years old (range 5-74 years) of *Aggregatibacter aphrophilus* endocarditis reported in the medical literature ^3^^-^^22^. The data on gender, age, clinical features, diagnostic tests, surgical treatment, and survival are summarized in Table 1.

### Underlying diseases and risk factors

A combination of the previous medical history of prosthetic valve, pacemaker placement, congenital heart disease, congenital valvular disease, prior rheumatic fever, poor dentition, chronic disease, drug abuse, and tongue piercings has been reported in the majority of the cases. Only six patients (30%) did not present risk factors.

According to previous reports, five patients (25%) had received a prosthetic valve. One patient had undergone aortic valve replacement (AVR) twice due to infective endocarditis (IE) and a subsequent failed bioprosthetic valve; a second patient had a bioprosthetic aortic valve replacement at the age of 17 for bicuspid aortic stenosis; a third patient had undergone AVR twice and a mitral valve replacement (MVR) at 51 years old, also, the patient presented rheumatic fever at 15 years old; he was being treated with beta-blocker (atenolol) and a vitamin K antagonist (acenocoumarol); the fourth patient had undergone AVR due to a bicuspid aortic stenosis, also the patient presented diabetes mellitus; and the fifth patient had rheumatic fever.

Only one case (5%) received a dual-chamber pacemaker placement for complete heart block. A 25% of the patients presented with congenital valvulopathy: true bicuspid aortic valve and ascending aortopathy were reported in one of the patients; while the second patient had a calcific aortic stenosis due to a congenital bicuspid aortic valve and a poor dentition. The third patient had a pierced tongue two months before onset of illness and a history of aortic valvuloplasty at eight years of age for correction of congenital aortic stenosis. Also, the patient had previous dental work with endocarditis prophylaxis. Among other reported conditions, one of the patients had a calcific aortic stenosis due to a congenital bicuspid aortic valve and a poor dentition.

Three cases (15%) had congenital heart disease. One patient had a perimembranous ventricular septal defect (PMVSD); a second patient had a Contegra D-valved conduit (CVC) placement due to a D-transposition of the great arteries with ventricular septal defect and pulmonary stenosis at two years old; a third patient underwent a surgical patch closure of patent foramen ovale at the age of 9 and dental care at five months before his admission. Only one case (5%) had a history of nicotine and alcohol abuse.

### Clinical presentation and physical examination 

The initial presentations of 20 patients with endocarditis due to *Aggregatibacter aphrophilus* were detailed. The mean duration of symptoms before diagnosis in 16 patients was 10 days (range, 5 - 14 days). The clinical presentation was available for 19 patients. The most common symptoms were fever in 16 (80%), fatigue/general malaise in 5 (25%), weight loss in 5 (25%), and headache in 3 (15%) patients. On the physical examination, cardiac murmurs were found in 6 patients (30%). A total of seven patients (35%) showed embolic complications as initial presentation, neurological involvement being the most common. Four patients (25%) had an ischemic stroke; 1 patient presented a brain abscess; 1 splenic abscess and 1 ANCA-positive glomerulonephritis were also described. Two patients (10%) were admitted with the initial diagnosis of heart failure. 

### Diagnosis

In 20 cases for which data were recorded, the mean positive blood culture was 0.59 (range: 1-8 taken) with a mean incubation time of 5 days (range: 3-7 days). In 8 patients, blood cultures yielded no organisms, but a definitive diagnosis of endocarditis was established by PCR/sequencing (Br-PCR) of the 16S ribosomal RNA gene in the resected valve or arterial embolus or by culture of the valve in surgery. In 1 case, *Aggregatibacter aphrophilus* was identified in the cerebrospinal fluid culture.

An echocardiogram was performed on 19 patients, of whom 6 underwent Trans thoracic echocardiogram (TTE) and 4 Trans-esophageal echocardiogram (TEE). In one patient, 2D-TEE, 2D-TTE and 3D-TTE were performed, of which only a positive result was obtained through 3D-TTE; 8 patients had both a TTE, and a TEE. In five patients, the vegetations were visible on the TEE, but not on the TTE. The size of the vegetations, determined by echocardiography, was described in only 8 cases. The mitral valve was involved in 8 of the 20 (40%) patients, the aortic valve in 1 (5%) patient, and both valves in 1 (5%) patient. One case of ventricular pacemaker lead infection was presented. In 9 patients (45%) the valve involved was not identified.

### Treatment and susceptibility

The treatment of the 20 patients was detailed, all of whom received cephalosporins at some point during the course of therapy. The therapy was almost always administered intravenously. The most frequently administered therapy was cephalosporin monotherapy (10 patients, 47.6%) followed by dual cephalosporin and aminoglycoside therapy (3 patients, 15%). One patient received the combination of a cephalosporin plus a glycopeptide, while other regimens included tetracyclines, rifamycins, and penicillins. One patient received a cephalosporin, a fluoroquinolone, a glycopeptide, and 3 other antimicrobial agents. Cephalosporins were part of the antimicrobial therapy in 17 (89.5%) cases. The mean duration of treatment in 12 patients was 4.9 ± 6 weeks (range: 2 weeks to 8 weeks). The median duration of treatment for native valve endocarditis was 2.6 weeks and for prosthetic valve endocarditis 3.6 weeks; 50% of the patients (10) underwent valve replacement surgery.

### Outcome

Complications included ischemic stroke in 4 patients (20%), glomerulonephritis in 1 patient (5%), brain abscess in 1 patient (5%), and splenic abscess in 1 patient (5%). Nineteen of twenty patients (95%) were cured; the outcome was not specified for 1 case. In cases related to native valves, valve replacement was required in 4 (20%) patients; 2 (33.33%) of the 6 patients with involvement of the prosthetic valve required valve replacement. Of the 4 cases of native valve endocarditis, the aortic valve was replaced in 1 and the mitral valve in 3 patients. Of the 6 cases of prosthetic valve endocarditis, 2 (33.33%) required aortic valve replacement.

## Discussion

*Aggregatibacter aphrophilus* is a member of the group of HACEK organisms*.* Typically, *Aggregatibacter aphrophilus* is part of the normal oropharyngeal flora and is frequently found in dental plaques and gingival scrapings ^1^. Khiarat *et al.* described the first case of valvular *Aggregatibacter aphrophilus* infection in 1940 ^23^. *Aggregatibacter aphrophilus* is an uncommon cause of EI (1-3%). The highest incidence of *A. aphrophilus* endocarditis is among middle-aged adults and preferentially infects males ^2^. It is believed that the microorganism located in the oropharynx, enters the vascular chamber at the time of dental work or in the context of periodontal disease, normally in patients with poor dentition or recent dental work ^1^.

Therefore, the literature data suggest that the microorganism is generally considered to be low virulence and structurally damaged, or prosthetic cardiac valves seem to be the predisposing conditions most strongly associated with the incidence of *Aggregatibacter aphrophilus* endocarditis. Other groups at risk include those with pacemaker placement, congenital heart disease, prior rheumatic fever, poor dentition, chronic disease, drug abuse, and those with tongue piercings ^1^. 

*Aggregatibacter aphrophilus* endocarditis is remarkably insidious in its presentation ^8^. The course of symptoms before the diagnosis has been reported to be prolonged, with a mean of 10 days, compared to endocarditis caused by traditional organisms ^24^^,^^25^. Systemic symptoms, fever, weight loss, and anorexia were reported in most cases; however, embolic complications stood out as the initial clinical presentation. Embolic neurological involvement is the most common. The most reported conditions were cerebrovascular accidents and brain abscesses, patients can also have splenic infarction and other extracardiac emboli complications. The mitral valve is the most commonly infected valve, with a tendency to infect normal valves more often than other microorganisms do ^7^. The presence of factor V on its structure is necessary for the infection of the native valve ^26^.

The diagnosis is extraordinarily challenging ^27^. Knowing that the identification of the pathogen is the key to the success of the treatment of the endocarditis with HACEK organisms the problem is that they are well known as culture negative. It is currently suggested that the PCR/sequencing study (Br-PCR) of the 16S ribosomal RNA gene overcomes the difficulty of finding this microorganism in a blood culture. The diagnosis of *Aggregatibacter aphrophilus* endocarditis with the modified Duke criteria has limitations ^28^. The median number of cultures taken was 2.1 (range, 1-8 taken), of which 47% were positive for *Aggregatibacter aphrophilus* with a mean incubation time of 5 days (range, 3-7 days). In 8 patients, no organisms were isolated in the blood cultures, even though despite the fact that serial samples of more than 3 blood cultures were taken, separated by 24 hours each with an interval between samples of 60 minutes. *Aggregatibacter aphrophilus* needs to be considered as difficult organisms to culture and, therefore, they are classified within the group of “culture-negative endocarditis” ^3^.

For the diagnosis of endocarditis, the identification of vegetation on the heart valve was made principally by a transesophageal echocardiogram. Most patients who had an TEE report a previous negative transthoracic echocardiogram. Normally the first exam is the TTE, but in cases where vegetation cannot be observed, the primary second-line examination is a TEE. In our review, we identified that the vegetation was identified in 13 (65%) of the 20 patients using transesophageal echocardiography; of which 8 presented a negative initial transthoracic echocardiography. 

The American Heart Association (AHA) and European Society of Cardiology (ESC) recommend as a first-line treatment with intravenous third or fourth-generation cephalosporins and fluoroquinolones ^27^. Of the 20 cases presented, 17 used ceftriaxone as central treatment, 8 of which used only monotherapy with a third-generation cephalosporin for a mean of 4 weeks (range 2-8 weeks). Eight patients used double therapy where fluoroquinolones were used in 60%. In 10 of the 20 patients, the condition resolved after 6 weeks of antibiotic therapy without the need for surgical intervention. The routine duration of treatment is four-weeks for non-valvular endocarditis (NVE) and six-weeks for prosthetic-valve endocarditis (PVE). Patients with endocarditis due to *Aggregatibacter aphrophilus* achieve resolution of the condition through antibiotic therapy, valve replacement surgery is not frequent. Valve replacement surgery was necessary for 5 patients (25%), the aortic valve was replaced in 2 patients, and the mitral valve in 3 patients. No perioperative complications were reported. 

Endocarditis secondary to HACEK organisms generally has an excellent prognosis with a significantly lower mortality rate at one year compared to IE due to EGV ^13^. Most of the patients did not report complications, death, or recurrence of a new episode at follow-up for 1 year.

The review highlights the importance of a high index of suspicion in symptomatic patients with an initial negative blood culture as a *Aggregatibacter aphrophilu*s endocarditis, particularly in high-risk groups such as patients with congenital valve disease and prosthetic valve. The knowledge of this rare entity may lead to early diagnosis and appropriate management.
